# 

**Published:** 1880-03

**Authors:** 


### INDEX TO VOLUME XVII.

| | PAGE. |
|---|---|
| A Physiological Curiosity...... | 766 |
| Atlanta Academy of Medicine, ..............7, 67, 144, 198, 456, | 517 |
| Anesthesia, Discoverer of....... | 27 |
| Abortive Treatment of Bubo... | 34 |
| Aconite as a Therapeutical Agent.............................. | 40 |
| Alcoholism, Treatment of...... | 56 |
| American Medical College Association.............................. | 83 |
| American Medical Association | 109 |
| An Agreeable Surprise........... | 123 |
| A Manual of Examinatiou of the Eyes....... .................... | 123 |
| Atlas of Skin Diseases.......... | 125 |
| Abscess of the Tonsils........... | 127 |
| Alcoholic Dressing of Wounds | 234 |
| A Summary of Ferrier's Localization.............................. | 237 |
| A Treatise on the Horse and his Diseases...................... | 248 |
| American Health Primers...... | 248 |
| Academy of Medicine............ | 210 |
| American Gynæcological Society.............................. | 247 |
| A Text Book of Physiology... | 309 |
| A Medical Contract in Italy... | 320 |
| A Summer Madrigal............. | 320 |
| An Elephant's Gratitude........ | 372 |
| Anti-Toothache......... ........... | 375 |
| Action of Iodoform................ | 379 |
| A New Antiseptic................. | 380 |
| A Case of Long-Continued Priapism, etc......................... | 369 |
| Alleghany County Medical Society.............................. | 392 |
| Antiseptic Surgery............... | 420 |
| Analysis of the Urine, etc...... | 512 |
| American Public Health Association.............................. | 460 |
| Automatic Medical Electricity | 576 |
| Absorbtion.......................... | 623 |
| Advancement in Gynæcology.. | 643 |
| Ano-Rectal Abscess.............. | 647 |
| Atlanta Medical College......... | 682 |
| A New Anthelmentic............ | 693 |
| A System of Medicine........... | 689 |
| Application of Plaster of Paris Bandage.............................. | 700 |
| Aspirator in Retention of Urine | 751 |
| Bubo, abortive treatment of.... | 34 |
| Bulletin of the Public Health.. | 54 |
| Burns and Erysipelas, treatment of.............................. | 139 |
| Buffalo Medical Association... | 526 |
| Biographical Dictionary......... | 633 |
| Batley's Operation in Germany | 641 |
| Baltimore Academy of Medicine.............................. | 654 |
| Bursa Pastoris...................... | 698 |
| Chloroform.......................... | 764 |
| Congregation of Medical Men in Atlanta........................ | 51 |
| Clinical Lectures on Diseases Peculiar to Women........... | 47 |
| Capsicum and Quinine......... | 60 |
| Counter Practice in France.... | 60 |
| Cement for Wood and Iron...... | 61 |
| Cholera Infantum................. | 63 |
| Case of Retained Menstrual Matter, etc........................ | 65 |
| Convention of American Medical Colleges...................... | 77 |
| Cincinnati Medical Society..... | 152 |
| Canthotony for the Relief of Blepharopasm.................... | 193 |
| Chloric Acid........................ | 234 |
| Contributions to the Pathological Anatomy of Acute Delirium...................... | 318 |
| Chloral Poisoning, Nitrite of Amyl in........................... | 313 |
| Colic, treatment of............... | 315 |
| Compound Tincture of Iodoform.............................. | 384 |
| Cough Mixture.................... | 384 |
| Croton Oil in Vavus............ | 366 |
| Consumption ...................... | 344 |
| Clinical Lecture on Facial Palsy.............................. | 440 |
| Chicago Medical Society...... | 460 |
| Carcinoma Ventriculi........... | 489 |
| Clinical Lecture................... | 530 |
| Cysticercus Cellulosæ............ | 458 |
| Case of Insufficiency of Mitral Valves.............................. | 570 |
| Celluletis of the Neck........... | 577 |
| Citron Juice........................ | 578 |
| Carbolic Acid...................... | 580 |
| Compression and Immobilization, etc.............................. | 600 |
| Chryophanic Acid................ | 638 |

PAGE.
Changes of Muscle-Irritability, etc ........ 702
Diabetes, Table Diet for......... 35
Dyspepsia, treatment of...... 59
Dyptheria........ 62
Diseases of Live Stock........... 49
Diseases of the Intestines and Perineum ........ 247
Detroit Academy of Medicine 336
Diseases of Women........ 511
Do Nostrums Benefit Consumers........ 506
Double Vagina and Cervix Uteri........ 492
Diphtheria, treatment of......... 497
Dangers from Turpentine Vapor........ 573
Dysmenorrhœa, prescription for........ 573
Dyspepsia........ 652
Diagnosis of Tobacco Ambliopia........ 669
Eye and Ear Infirmary, etc.... 63
Erysipelas and Bruns, treatment of........ 139
Elements of Modern Chemistry 246
Editorial Correspondence, 303, 508, 563
Effective Cathartic........ 367
Errata........ 445
Exsection of Sup. Maxilliary Nerve........ 449
Electricity a Paralyzing Agent 479
Elegant Process of Capsuling Bottles........ 700
Fistula in Ano, case of.......... 161
Facial Palsy, clinical lecture on........ 440
Fibroid Uterus........ 451
First Lines of Therapeutics... 632
Formulary........ 706
For Baldness ........ 696
Gonorrhœa........ 64
Goitre, Surgical Treatment of
Gonorrhœa........ 765
Curjun Balsam in........ 63
Ginger Beer........ 373
Guide to the Examination of Urine........ 361
Gunshot Wound of Uterus..... 409
Gynæcologists........ 578
Glioma of Retina........ 754
Handbook of the Practice of Medicine........ 47
Health, and How to Promote It........ 124
Health Primers........124, 632, 691

PAGE.
Hahnemann and Homœopathy 188
Habitual Constipation........... 250
How to Preserve Cider........... 374
Hypodermic Injection of Morphia........ 360
Head Injury, etc........ 322
How to Prevent Mammary Abscess........ 447
History of Massage........ 446
Highland Malarial Fever...... 453
Hay Fever........ 639
Health, a Ministry of, etc...... 632
Hemorrhages from the Lungs.. 604
How to Grasp the Pelvis, etc.. 705
Hepatic Colic........ 701
Hydrate of Chloral........ 761
In Memoriam......51, 123, 629, 685
Information Wanted........ 62
Intestinal Obstruction, rare form of........ 126
Injections of Linseed Oil in Chronic Cystitis........ 366
Intravenous Injection of Ammonia........ 416
Inflammation of the Pleura... 435
Ipecac as a Hæmostatic......... 574
Liver, Clinical Treatise on Diseases of........48, 185
Long, Dr. C. W........ 27
Lithotomy, case of........ 174
Lactopeptine ........ 255
Lessons in Gynæcology......... 308
Lumbago ........ 362
Laryngitis Catarrhalis........... 515
Metrical System ........ 256
In Great Britain........ 58
Meterological Report, 122, 184, 249, 302, 377, 562, 634, 692, 762
Medical Education........ 180
Meningenal Hemorrhage, case of........ 169
Malpractice ........ 245
Mode of Securing Medical Education ........ 242
Malignandy in Tumors......... 212
Manual of the Principal and Practice of Operative Surgery ........ 307
Materia Medica and Therapeutics........ 362
Molar Pregnancy........ 349
Mixture of Digitalis, etc......... 446
Medical Teaching ........ 445
Manuary Abscess........ 575
Medical Chemistry ........567, 633
McDowell, E........ 632
Menthol........ 642

PAGE.
Membranous Croup, etc.......... 636
Malarial Fever, cause of........ 635
Morbid Impulses.................. 596
Medical and Surgical Society of Baltimore...................... 733
Nervous Exhaustion.............. 763
New Uterine Dilator.............. 5
National Board of Health...90, 757, 684
New York Academy of Medicine.................................. 145
Necrosis.............................. 222
New York Pathological Society 280, 462
Nitrate of Amyl in Chloral Poisoning ........................ 313
Nervous System, Diseases of... 309, 511
Neglect and Value of Blistering.......................................... 365
Nichols' Elixir...................... 512
Nitrate of Amyl in Uterine Hemorrhages..................... 697
Obstetrics, Case of............... 1
Obstinate Vomiting............... 364
Organic Disease of Heart...... 738
Patent Medicines.................. 57
Practical Gynæcology........... 46
Practitioners' Reference Book 46
Pamphlets Received....49, 125, 187
Paper on Quarantine............. 74
Psoriasis.............................. 165
Poisoning by Carbolic Acid ... 256
Posological Table ................. 248
Poisoning ............................ 319
Plugging the Nasal Cavities... 320
Pocket Therapeutics and Dose Book.................................... 307
Physiological Antagonism the Therapeutic Law of Cure.... 284
Physiology of the Secretion of Sweat................................... 291
Pilocarpinum Muriaticum in Children's Diseases .......316, 383
Philadelphia County Medical Society ...........................353, 593
Professional Honorarium....... 359
Physiology and Histology of the Cerebral Convolutions. . 361
Pilocarpin in the Pruritis of Pregnancy............................ 368
Proceedings Cincinnati Academy of Medicine............... 394
Pathology and Treatment of Ulcers, etc........................... 472
Phosphorole......................... 512
Post nasal Catarrh................ 546

PAGE.
Pertussis, Treatment of.......... 574
Pseudo Tumor of Abdomen... 611
Proceedings Cincinnati Medical Society......................... 660
Paracentisis of the Pericardium................................... 691
Pilocarpin in Uræmia........... 694
Puerperal Fever.................... 696
Physiological Experiments..... 706
Palatable Castor Oil.............. 706
Quarantine, Paper on............ 74
Quinine Rash........................ 321
Quarantine an Advertiser...... 332
Quinia and Amyl Nitrite in Pertussis............................ 568
Quinine Test......................... 698
Religion and Chloroform........ 64
Removal of Steel Rust........... 64
Recent Dermatological Literature upon "New Formations" 129
Remarkable Sympathetic Disturbance............................. 255
Resuscitation from Chloroform Syncope.............................. 195
Rubeola and its Treatment..... 257
Red Cinchona....................... 378
Regeneration of the Eye........ 448
Reflex Nervous Troubles, etc. 387
Recent Progressive Treatment of Mental Diseases ............ 553
Remarkable Menstruation...... 575
Russian Gargle...................... 578
Spanked...... ........................ 61
Suffolk District Medical Society..................................... 15
Sympathetic Insanity............ 37
Stricture of Female Urethra... 142
State Medical Association...... 183
Spermatorrhœa..................... 186
Sweat-Producing Agents........ 252
Salycilic Acid, etc................. 382
Sclerotinic Acid in Hæmoptysis 367
Salycilic Acid as an Anti-Scorbutic.............................. 578
St. Louis Medico Chirurgical Society ............................... 518
Sciatica Relieved by Galvanism 579
Some Affections of the Upper Air Passages.................... 614
Sweating, Physiology of......... 640
Sixth Decennial Pharmacopœia Convention................ 688
Select Topics for Modern Surgery.................................... 672
St. Louis Obstetrical Society... 720
Spontaneous Rupture of Uterus 748
The Largest Infant on Record 58

PAGE.

Tax on Doctors.................... 43
Toledo Medical Association.... 14
The Tapeworm...................... 128
Therapeutics, Treatise on...... 185
The name "Allopath"........... 251
The American Association for the care of Inebriates......... 203
Treatment of Colic................ 315
Theories of Diseases.............. 293
The Medical Society of the County of New York......... 267
The Classification of Medicine 381
The Therapeutic Value of Croton Chloral.................. 371
Treatment of Indolent Ulcers.. 368
The Essential Physiology of Respiration...................... 324
The Communicable Fevers and their Treatment........... 342
The Fever of Neighboring Counties.......................... 444
Theories of Fever................. 442
Toledo Medical Association.... 399
Trismus Nascentium ............ 385
The National Dispensatory..... 446
The Multum in Parvo Reference Book..................... 512

PAGE.

The Treatment of Diseases by the Hypodermic Method..... 76
The Immediate as Distinguished from the Permanent Treatment of Disease......... 569
Thymal Soap........................ 575
Treatmeut of Tetanus.............. 620
Therapeutics of Gynæcology.. 689
The Hypodermic Injection of Morphia ........................ 690
Treatment of Bursa Patellæ Amplificata ...................... 695
Test for Quinia Sulphate........ 699
The Benzoates...................... 702
Treatment of Asthma............ 706
The Bandage, etc.................. 707
The Microscope and Microscopical Technology........... 763
Urinary Calculi..................... 378
Urticuria............................ 639
Vomiting of Pregnancy...265, 370
Vaccination......................... 641
Valedictory Address.............. 715
Walsh's Physicians' Ledger.... 631
Yellow Fever.....................48, 300
A Nautical Disease......... 567

